# Characterization of small-scale net fisheries off the coast of Guyana

**DOI:** 10.1371/journal.pone.0306332

**Published:** 2024-06-28

**Authors:** Rovindra Lakenarine, Netra Chhetri, Neha Chhetri, Jesse Senko

**Affiliations:** 1 School for the Future of Innovation in Society, College of Global Futures, Arizona State University, Tempe, Arizona, United States of America; 2 Department of Biology, Faculty of Natural Sciences, University of Guyana, Georgetown, Guyana; 3 School of Ocean Futures, College of Global Futures, Arizona State University, Tempe, Arizona, United States of America; Ocean Frontier Institute, CANADA

## Abstract

Fish stocks have declined rapidly over the past half-century due to the increased demand for seafood and unsustainable fishing practices. The incidental capture of non-target species (bycatch) is a pervasive issue in fisheries management and has led to population declines in non-target species worldwide. The fisheries sector in Guyana currently supports the livelihoods of over 10,000 Guyanese and contributes approximately 2% to the country’s GDP. Bycatch is believed to be a major threat to Guyana’s marine fisheries, especially the small-scale sector, due to a lack of management infrastructure and limited data and monitoring. Here, we assessed bycatch in Guyana’s artisanal gillnet and Chinese seine fisheries through vessel observations and semi-structured interviews with local fishers. Most of the discarded species documented had no commercial importance to the fisheries in Guyana. Although no statistical difference was observed among the bycatch rates in the gillnet and Chinese seine fisheries, the latter generally had more discarded individuals, most of which were juveniles. The Shannon-Weiner diversity index showed a greater diversity of bycatch species in the gillnet fisheries compared to the Chinese seine. Jaccard’s similarity index indicated a low similarity among the gear types. Even though most fishers were aware of bycatch, they did not view it as a major issue and were not interested in reducing their discards. We recommend a collaborative approach in exploring solutions to ensure the ecological and socioeconomic sustainability of the fisheries sector.

## 1. Introduction

Globally, over three billion people depend on marine and coastal resources for their livelihoods [1]. Marine fisheries directly or indirectly support the livelihoods of over 200 million people worldwide, and fish products are a major protein source for billions of people [2, 3]. Over 80 million tons of marine fish are harvested for consumption annually [4]. However, the world’s marine resources are under increasing pressure due to the rapidly rising demand for seafood products [5].

Research suggests that 70% of the world’s marine fish stocks are over or fully exploited [6]. Many capture fisheries across the globe discard more fish than they retain, while over 11% of the world’s population is undernourished and lacks reliable protein sources [7]. As such, there is an urgent need to understand how fisheries will respond to growing pressures while maintaining marine biodiversity, food security, livelihoods, and resilient economies, particularly in developing countries.

One of the major issues the global fisheries sector faces is bycatch [8], the unintentional capture of non-target species during fishing operations [9]. Over the last 20 years, bycatch has become a major challenge for managing global fisheries [8, 10]. The estimated global bycatch amounts to approximately 29 billion kilograms per year, or 40% of the world’s total catch [11, 12].

Bycatch monitoring and mitigation is a pervasive issue for artisanal fisheries worldwide. The lack of data on the amount and composition of bycatch species makes managing artisanal fisheries in developing nations challenging [11–13]. While successful mitigation measures have been developed in industrial-scale fisheries, such as turtle excluder devices (TEDs) [14] and the Nordmore grid [15], we lack a comprehensive understanding of bycatch impacts across most artisanal fisheries [9, 13].

The fisheries sector in Guyana is an essential source of employment, food, and revenue for coastal communities throughout the country [16–18]. The industry contributes approximately 2% of Guyana’s GDP and employs over 11,000 people [16]. The key marine fisheries sectors in Guyana are the industrial, semi-industrial, and artisanal fisheries. The industrial fishery or trawl fishery targets seabob (*Xiphopenaeus kroyeri*) and prawns (*Penaeus brasiliensis*, *Penaeus notialis*, *Penaeus schmitti*, and *Penaeus subtilis*), while the semi-industrial fishery targets vermilion snapper (*Rhomboplites aurorubens*), and red snapper (*Lutjanus purpureus*) [17]. The artisanal fishery is the largest, consisting of over 1,300 vessels, with the most common gear types being gillnets and Chinese seine nets [17]. The common target species are sea trout (*Cynoscion virescens*), bangamary (*Macrodon ancylodon*), grey snapper (*Cynoscion acoupa*), gillbacker (*Sciades parkeri*), catfish (*Bagre bagre*), seabob (*Xiphopenaeus kroyeri*), and butterfish (*Nebris microps*) [17]. We examined the finfish bycatch composition of the gillnet and Chinese seine fisheries, the conservation status of the bycatch species, and fishers’ awareness of bycatch in Guyana.

## 2. Method

### 2.1 Study site

Vessel observations were conducted at two landing sites for each gear type. The Chinese seine vessel observations were done in Administrative Region Four at the Ogle Landing Site and Administrative Region Five at the Rosignol Landing Site. The primary target species for the Chinese seine fishery were eabob (*Xiphopenaeus kroyeri*) and whitebelly shrimp *(Nematopalaemon schmitti)*. The gillnet vessel observations were done at Hope landing site in administrative region four and De Edward Koker Door #3 in administrative region five. The gillnet fishery primarily targets bangamary (*Macrodon ancylodon*) and butterfish (*Nebris microps*). Fisher surveys were conducted at the Ogle, Liliendaal, Hope, and Emore landing sites in administrative region four and at De Edward Koker Door #3, Mahaicony, and Rosignol landing sites in administrative region five. The study sites selected are major fish landing sites for the gear types within the fishing regions selected. The two administrative regions selected are also major fishing regions in Guyana.

### 2.2 Quantitative data collection

Onboard observations were conducted on two small-scale Chinese seine and two gillnet vessels from the two study regions following the method used by [13, 19, 20] between August 18, 2021 –September 30, 2021. The most productive fishing period in Guyana is between March and October. The fishers collected all the bycatch and brought them onto shore for examination. The vessels were chosen based on the owners’/captains’ willingness to participate in the research and confirmation that they are registered boat owners with the fisheries department in Guyana. Data was collected from each vessel for the duration of the fishing trip, for three fishing trips each, typically lasting 4–6 hours for Chinese seine vessels and 4–8 hours for gillnet vessels. The Chinese seine nets had a mesh size ranging from 6" at the mouth to 0.5" at the funnel (tapered end) with a length of approximately 18–30 m. By contrast, gillnets had a mesh size of 2.75"– 3" with a length of approximately 3218 m. The time of setting and retrieving the seine was recorded for each haul throughout the data collection period for the Chinese seine and gillnet. This was used to calculate the soak time, the duration between setting and retrieval.

The unsorted catch was weighed for each haul using an analog scale. The bin’s weight was extracted from the overall total to determine the total catch. The fishers then sorted the catch to separate the target and bycatch species. A physical count of all individual bycatch species was taken along with their collective weight. This weight was subtracted from the total weight of the catch to estimate the bycatch. This was used to calculate the bycatch rate or bycatch per unit effort (BPUE), calculated as BPUE = kg bycatch species/ ([net length/100m] x [net soak time/12h]) [21]. A random sample of 20 individuals of each species from each net was measured (standard length) and weighed (to the nearest kg). The target fish rate or catch per unit effort (CPUE) was also calculated for each gear as CPUE = kg of target catch/ ([net length/100m] x [net soak time/ 12h]) [21]. The catch per unit effort (CPUE) represents the commercial catch rate per unit of fishing effort (the length of the net and soak time).

Each bycatch fish species was photographed and identified using fish identification guides. Unidentified specimens were collected and stored in 10% formaldehyde and then transferred to 95% ethanol for taxonomic confirmation and permanent storage at the Center for the Study of Biological Diversity, University of Guyana, Guyana. The conservation status of each bycatch species was evaluated using the IUCN red list.

### 2.3 Qualitative data collection

The random sampling technique was used to collect general perception data from fishers through semi-structured interviews. We randomly surveyed fishers from 15% of vessels for each gear type present across landing sites in regions four and five between August 18, 2021—September 30, 2021. The captain of each selected vessel was interviewed. The participants were interviewed immediately after recruitment in a semi-private space at the landing sites. They were briefed on the study, and oral consent was required before participating. Questions asked were: “During your fishing trips, do you catch any species, other than those that were not targeted, that you tossed back/discarded?” “In your opinion, what is bycatch?” “In your estimation, what percentage of your total catch is discarded (trash)?” “What impacts do you think bycatch (trash/mess) may have on your fishing activities?” “Are you interested in reducing your discards/trash?” “Do you think there are types of fishing gear that should be restricted?”

### 2.4 Data analysis

The quantitative data were used to determine species diversity and abundance of discards, and a species composition table was generated. The Shapiro-Wilk normality test was used to check for normality among the data. The species composition from the different gear types was compared using the Shannon—Weiner Diversity Index. The Shannon—Weiner Diversity Index is used to estimate species diversity. It considers the species richness and evenness [22]. Jaccard’s similarity index was used to measure the similarity between two data sets (bycatch species in the Chinese seine and gillnet fisheries) [23]. A Kruskal Wallis test was also used to determine the statistical differences between BPUE and CPUE for the Chinese seine and gillnet. The qualitative data were examined to determine fishermen’s perceptions and were analyzed using simple descriptive analysis.

### 2.5 Ethics statement

This study protocol was approved by the Arizona State University Institutional Review Board (approval number: 00014576). Verbal consent was obtained from fishers before participating in the study.

## 3. Results

### 3.1 Bycatch species composition

**Fig 1:**
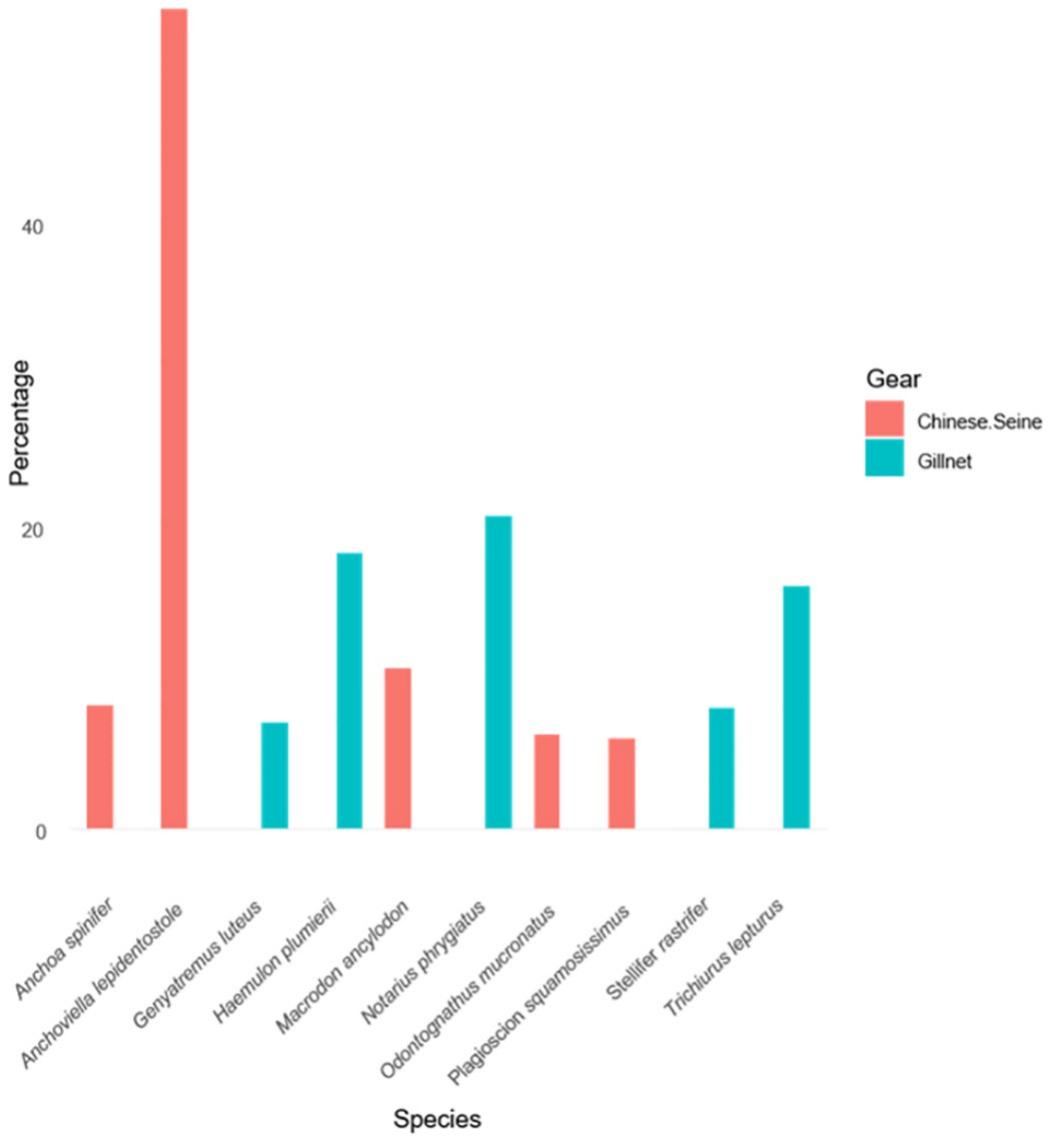
Overall bycatch species composition for the five most abundant species in the Chinese seine and gillnet fisheries.

Forty-nine species (Number of Individuals (N) = 21,447) from eleven orders and twenty-five families were discarded from the Chinese seine and gillnet fisheries. Fifty-seven percent of the species had no commercial importance to the fisheries in Guyana, while 43% had some commercial importance (Table 1). Additionally, 14% of the species were used for recreational purposes, while 86% had no recreational importance (Table 1). Perciformes was the most abundant order (Relative Abundance (RA) = 51%), Sciaenidae the most abundant family (RA = 18%), and *Anchoviella lepidentostole* (Suriname mullet) the most abundant species (N = 11493, RA = 54%) (Table 1). There were more discarded species in the Chinese seine fisheries. Thirty-five species (N = 21,147) were discarded in the Chinese seine fishery, while 28 (N = 300) were discarded in the gillnet fishery. *Anchoviella lepidentostole* (Suriname mullet: N = 11,493, RA = 54.3%) recorded the greatest number of discarded individuals for the Chinese seine vessels and *Notarius phrygiatus* (Thomas/ sea catfish: N = 62, RA = 20.7%) for the gillnet (Table 2, Fig 1). The majority (76%) of the species were listed as ’least concern’ on the IUCN red list of threatened species; however, one species (*Carcharhinus porosus*–waterbelly shark, N = 7) was listed as critically endangered (Table 1).

**Table 1:** Summary of selected characteristics of the bycatch species recorded from the gillnet and Chinese seine vessels.

Order	Family	Species	Local Name	n	IUCN Status	Value to Guyana’s Fisheries	
						Com	Rec
**Anguilliformes**	Congridae	*Conger triporiceps*	Sea eel	3	LC	✓	X
**Beloniformes**	Belonidae	*Strongylura marina*	Swordfish	5	LC	X	✓
**Carcharhiniformes**	Carcharhinidae	*Carcharhinus porosus*	Water belly shark	7	CR	✓	✓
		*Carcharhinus sp*.	Boraman shark	1		✓	
**Clupeiformes**	Clupeidae	*Odontognathus mucronatus*	Netley	1312	LC	✓	X
	Engraulidae	*Anchoa spinifer*	Brown Herring	1734	LC	X	X
		*Cetengraulis edentulus*	Par / Atlantic anchoveta	479	LC	X	X
		*Lycengraulis grossidens*	Silver herring	24	LC	X	X
		*Anchoviella lepidentostole*	Suriname mullet	11493	LC	✓	X
	Pristigasteridae	*Pellona harroweri*	Overrun	19	LC	X	X
		*Pellona sp*.	Watermelon	5		X	X
**Mugiliformes**	Mugilidae	*Mugil curema*	Mullet	5	LC	✓	X
**Perciformes**	Centropomidae	*Centropomus pectinatus*	Chinese Snook	3	LC	✓	X
		*Centropomus ensiferus*	Snook	1	LC	✓	X
	Carangidae	*Oligoplites saliens*	Copper fish	64	LC	X	X
		*Oligoplites palometa*	Copper fish	15	LC	X	X
		*Selene vomer*	Look down	4	LC	X	X
		*Trachinotu sp*.	Copper fish	15		X	X
		*Chloroscombrus sp*.	Copper fish	125		X	X
	Echeneidae	*Echeneis naucrates*	Remora	1	LC	X	X
	Ephippidae	*Chaetodipterus faber*	Donkeyfish/Atlantic spade fish	3	LC	X	X
	Gobiidae	*Gobioides broussonnetii*	Candle fish	25	LC	X	X
	Haemulidae	*Haemulon plumierii*	Grunt	55	LC	✓	✓
		*Genyatremus luteus*	Anafolks	66	DD	✓	X
	Lobotidae	*Lobotes surinamensis*	Paggy	4	LC	✓	X
	Stromateidae	*Peprilus paru*	Dollarfish	1	LC	X	X
	Sciaenidae	*Nebris microps*	Butterfish	17	LC		X
		*Stellifer rastrifer*	Rockhead	862	LC	X	X
		*Stellifer microps*	Rockhead	390	LC	X	X
		*Menticirrhus sp*.	Round mouth/ white belly	2		✓	✓
		*Plagioscion squamosissimus*	Shine Banga	1280	LC	X	X
		*Cynoscion virescens*	Trout	6	LC	✓	✓
		*Umbrina sp*.	Atlantic Croaker	2		✓	X
		*Macrodon ancylodon*	Bangamary	2273	LC	✓	X
		*Lonchurus elegans*	Chinese Butterfish	39	DD	X	X
	Scombridae	*Scomberomorus sp*.	Mackerel	20		✓	X
	Trichiuridae	*Trichiurus lepturus*	Silver belt	94	LC	X	X
**Pleuronectiformes**	Achiridae	*Achirus achirus*	Flounder	1	LC	X	X
**Siluriformes**	Ariidae	*Bagre Bagre*	Catfish	30	LC	✓	X
		*Sciades herzbergii*	Mudcuirass	259	LC	✓	X
		*Notarius grandicassis*	Thomas/ Sea Catfish	1	LC	✓	X
		*Sciades parkeri*	Gillbaka	1	VU	✓	✓
		*Notarius phrygiatus*	Kukwari	62	LC	X	X
		*Amphiarius rugispinis*	TweeTwee	457	LC	X	X
	Aspredinidae	*Aspredo aspredo*	Banjoman	24	LC	X	X
	Auchenipteridae	*Pseudauchenipterus nodosus*	Sweetman	68	LC	X	X
**Rhinopristiformes**	Rhinobatidae	*Pseudobatos percellens*	Guitar fish	1	EN	X	X
**Gymnotiformes**	Sternopygidae	*Sternopygus macrurus*	Knife fish	1	LC	X	X
**Tetraodontiformes**	Tetraodontidae	*Colomesus psittacus*	Puffer fish	88	LC	X	X]

*n = number of individuals/abundances

**DD—data deficient, LC—least concern, VU—vulnerable, EN—endangered, CR—critically endangered, NL—not listed

✓- Commercial/ Recreational Value X—No commercial/ recreational value

**Table 2:** Bycatch composition of the gillnet and Chinese seine vessels.

Species	Abundance			
	Chinese Seine		Gillnet	
	n	%	n	%
*Achirus achirus*	1	0.0	0	0.0
*Amphiarius rugispinis*	454	2.1	3	1.0
*Anchoa spinifer*	1727	8.2	7	2.3
*Anchoviella lepidentostole*	11493	54.3	0	0.0
*Aspredo aspredo*	24	0.1	0	0.0
*Bagre Bagre*	15	0.1	15	5.0
*Carcharhinus porosus*	0	0.0	7	2.3
*Carcharhinus sp*.	0	0.0	1	0.3
*Centropomus ensiferus*	0	0.0	1	0.3
*Centropomus pectinatus*	0	0.0	3	1.0
*Cetengraulis edentulus*	479	2.3	0	0.0
*Chaetodipterus faber*	1	0.0	2	0.7
*Chloroscombrus sp*.	125	0.6	0	0.0
*Colomesus psittacus*	88	0.4	0	0.0
*Conger triporiceps*	0	0.0	3	1.0
*Cynoscion virescens*	6	0.0	0	0.0
*Echeneis naucrates*	1	0.0	0	0.0
*Genyatremus luteus*	45	0.2	21	7.0
*Gobioides broussonnetii*	25	0.1	0	0.0
*Haemulon plumierii*	0	0.0	55	18.3
*Lobotes surinamensis*	3	0.0	1	0.3
*Lonchurus elegans*	39	0.2	0	0.0
*Lycengraulis grossidens*	23	0.1	1	0.3
*Macrodon ancylodon*	2272	10.7	1	0.3
*Menticirrhus sp*.	0	0.0	2	0.7
*Mugil curema*	5	0.0	0	0.0
*Nebris microps*	17	0.1	0	0.0
*Notarius grandicassis*	0	0.0	1	0.3
*Notarius phrygiatus*	0	0.0	62	20.7
*Odontognathus mucronatus*	1312	6.2	0	0.0
*Oligoplites palometa*	15	0.1	0	0.0
*Oligoplites saliens*	64	0.3	0	0.0
*Pellona harroweri*	0	0.0	19	6.3
*Pellona sp*.	0	0.0	5	1.7
*Peprilus paru*	0	0.0	1	0.3
*Plagioscion squamosissimus*	1274	6.0	6	2.0
*Pseudauchenipterus nodosus*	65	0.3	3	1.0
*Pseudobatos percellens*	0	0.0	1	0.3
*Sciades herzbergii*	256	1.2	3	1.0
*Sciades parkeri*	1	0.0	0	0.0
*Scomberomorus sp*.	18	0.1	2	0.7
*Selene vomer*	4	0.0	0	0.0
*Stellifer microps*	390	1.8	0	0.0
*Stellifer rastrifer*	838	4.0	24	8.0
*Sternopygus macrurus*	1	0.0	0	0.0
*Strongylura marina*	5	0.0	0	0.0
*Trachinotu sp*.	15	0.1	0	0.0
*Trichiurus lepturus*	46	0.2	48	16.0
*Umbrina sp*.	0	0.0	2	0.7

n = number of individuals

### 3.2 Diversity of Chinese seine and gillnet bycatch

The Shannon-wiener diversity index indicated a greater diversity of bycatch species in the gillnet fisheries (2.5) compared to the Chinese seine (1.7). The Jaccard’s similarity index showed a low similarity (29%) among the bycatch species of the two gear types.

### 3.3 Finfish bycatch per unit effort (BPUE) and catch per unit effort (CPUE)

The Chinese seine fisheries recorded higher bycatch and commercial catch rates. The bycatch rate was lower than the commercial catch rate in the Chinese seine and gillnet fisheries. The mean commercial catch rate of the Chinese seine was 59.1 (SD = ±24.6), while the mean commercial catch of the gillnet seine was 0.8 (SD = ±0.2). By contrast, the mean bycatch of the Chinese seine was 20.4 (SD = ±10.5), and the mean bycatch of the gillnet seine was 0.1 (SD = ±0.04). The Shapiro-Wilk normality test was used to check for normality among the data (BPUE and CPUE). The p-value was less than 0.05 (*W* = 0.759, *P* = 6.8e-0.5), so the null hypothesis of normal distribution was rejected. The Kruskal Wallis test showed no statistical difference (*P*>0.05) among the commercial catch of the gillnet and Chinese fisheries, bycatch among the gillnet and Chinese seine fisheries, commercial catch and bycatch of the Chinese seine fisheries, and commercial catch and bycatch of the gillnet fisheries.

### 3.4 Fishers perception of bycatch

While discards were recorded from all vessels and fishers indicated that they discard fish species, most fishers were unfamiliar with the term ’bycatch.’ They instead refer to bycatch as ‘mess,’ ‘trash,’ or ‘unwanted catch.’ These were fish that were considered non-marketable or undesirable.

The majority (77%) of Chinese seine fishers primarily target the White Belly Shrimp *(Nematopalaemon schmitti)* and Seabob (*Xiphopenaeus kroyeri*). On the other hand, the gillnet fishers’ primary targets are bangamary (*Macrodon ancylodon*) and butterfish (*Nebris microps*).

The Chinese seine fishers also capture bangamary, catfish (*Bagre Bagre*), and Cuirass (*Sciades herzbergii*) as secondary target species, while gillnet fishers collect trout and snapper *(Cynoscion acoupa)* as secondary target species. These species are kept to support an existing consumer market and home consumption. Much of the other species caught are usually discarded.

### 3.5 Fishery regulation

Most (88% Chinese seine and 83% gillnet) fishers indicated that the discarded species do not impact their fishing activities. However, some fishers believe that bycatch increases sorting time and damages the fishing gear. Most fishers (77% Chinese seine and 68% gillnet) were not interested in reducing discards. While the fishers indicated a decline in the fisheries, most (65%) of Chinese seine fishers and 37% of gillnet fishers indicated that no fishing gear should be restricted since the gears are not causing any major harm, there are no known alternatives and restrictions will decrease the profit of the fishers. Twenty-seven percent (27%) of the Chinese seine fishers and 61% of the gillnet fishers shared the view that some types of fishing gear, such as trawlers, should be restricted to ensure the sustainable fishing of targeted species and reduce habitat destruction, discards and conflicts among fishers.

## 4. Discussion

### 4.1 Bycatch species composition

The diversity of the bycatch species observed in both artisanal fisheries explored was consistent with previous studies and common knowledge that the artisanal fishery is of a multi-species nature [24]. There was also a greater number of discarded individuals recorded in the Chinese seine than in the gillnet fisheries. Over 99% of the individuals recorded were from the Chinese Seine fisheries. The lower number of species in the gillnet fisheries is possible because of the restricted eye size, targeting species of only a particular size. In contrast, Chinese seines have varying eye sizes and can capture more species [19, 20, 25]. The Chinese seine tapers into a bag at the end of the net to capture the shrimp; however, all the other finfish species that get into the net are also caught.

These trends were similar to those of [19], who compared finfish bycatch in the pin seine and Chinese seine fisheries and found a greater diversity and abundance of bycatch species in the Chinese Seine fisheries. The trends were also consistent with [20], who found over 32 finfish bycatch species from the Chinese seine fishery in the Demerara River, as well as [25], who found Chinese seine to have the highest discard rate when compared to three other gear types. Interestingly, the bycatch from the gillnet fishery recorded higher diversity indices than the Chinese seine fishery, even though the Chinese seine fishery had a greater number of individuals and species. This likely occurred because there was a more even distribution among the species captured by the gillnet fisheries compared to the Chinese seine, where a few species dominated the bycatch.

While the catch per unit effort (commercial catch) was greater than the bycatch per unit effort for both gear types, they were significantly lower in the gillnet fishery. The variation in the fishing effort, gears, and possibly environmental factors were potential reasons for the significant difference in the catch per unit effort and bycatch per unit effort for both gear types [19]. The Chinese seine fishers have shorter but more frequent fishing trips. On the other hand, the gillnet fisheries have longer but less frequent fishing trips and target larger fish.

The *Anchoviella lepidentostole* (Suriname mullet) was the most dominant species in the Chinese seine fishers and had the highest number of individuals overall. These species travel in schools in estuarine environments [26]. The fishers likely encountered schools of these fishes, which would account for the large numbers that were discarded. While this species was recorded as a common bycatch species in the Chinese seine fishery in Guyana, it was not the dominant species in other studies [19, 20]. Seasonality, water quality changes, and changing habitat dynamics might have resulted in this variation. By contrast, the *Notarius phrygiatus*, called Kukwari or Sea catfish, was the dominant species in the gillnet fisheries. These fishes are mainly found in marine and brackish water but are also encountered in freshwater environments [26]. Even though these species do not have a high market value, some of the bycatch is retained for food in Guyana.

### 4.2 Conservation status and conflicts

Most finfish bycatch was listed as least concerned on the IUCN red list, including the dominant species observed in the Chinese seine and gillnet fisheries. However, the number of discarded individuals is concerning in both fisheries. Further, while the length at maturity for species such as *Anchoviella lepidentostole* is 9.4 cm [26], the discarded individuals had a mean length and standard deviation of 6.2 ± 1.3, which suggests that most fishes were juveniles.

*Macrodon ancylodon* (bangamary) was recorded as the second dominant bycatch species in the Chinese seine fishery. This species was also listed as least concerned on the IUCN red list; however, the number of mature individuals in this population is decreasing and may be listed as a species of concern in the future [26, 27]. The length at maturity for this species is 23.7 cm [26]; however, most discarded individuals had a mean length and standard deviation of 8.4 ± 17.0. This is potentially problematic as almost all individuals recorded for this species were juveniles. This species is also of high commercial importance for Guyana’s local and export markets [17]. In fact, they are one of the main target species of the gillnet fishery, which may lead to conflicts between the two gear types since the Chinese seine fishers discard the majority of juveniles. This will likely decrease the target catch for the gillnet fishers, and it is an issue that needs to be monitored due to the vulnerability of this species [16].

Similar trends were also observed in the gillnet fishery; however, most bycatch individuals were observed to be adult or near adulthood. *Notarius phrygiatus* was the most dominant species in this fishery, and while the length at maturity is not listed for this species, the maximum length is 35.5 cm [26]. The mean length and standard deviation of the discarded individuals of this species were 30.2 ± 4.2, which is close to the maximum length of the species and suggests that most individuals were likely adults. The species was listed as least concerned on the IUCN red list [28]. *Haemulon plumierii*, commonly called grunt, was also recorded as a dominant discard species in the gillnet fishery. The length at maturity of the species is 18.3 cm, and the mean length and standard deviation of the discarded individuals of this species were 18.3 ± 1.2 cm, suggesting that most of the individuals were adults [26]. There is also no major conservation concern for this species; however, it is known to cause some form of ciguatera poisoning, which is probably why it is discarded [26]. On the other hand, *Pseudobatos percellens* (Guitar fish) was listed as endangered, and *Carcharhinus porosuson* (Water belly shark) was critically endangered on the IUCN red list. While only one individual of the *Pseudobatos percellens* was recorded, seven individuals of the *Carcharhinus porosuson* were documented, raising conservation concerns for this species.

Overall, the Chinese seine fishery poses a significant conservation concern as most of the species captured by this gear type were juveniles and in large numbers. This threatens species with late maturity as they will be highly vulnerable to extinction risks [29–31]. Most bycatch species are also school-forming, and their populations are more vulnerable to fishing gear, especially as they migrate [30].

### 4.3 Fishers perception and management of bycatch

The complexity surrounding bycatch mitigation can be attributed to the lack of awareness among the fishers. Understanding the problems of bycatch and developing practical solutions is a multi-disciplinary challenge that must consider the scientific, sociocultural, and socioeconomic components [32]. Many of these approaches are still developing as there remains a need to comprehensively understand fishers’ views. Workable solutions must be balanced as they should consider the ecological issue and the cost to fishers’ livelihoods as bycatch strategies are considered.

Small-scale fisheries play a key role in supporting the livelihoods of the rural population in developing countries [33]. The fisheries also alleviate poverty in communities as most of the population depend on fishing as their primary and sometimes only source of income [33]. A similar trend is seen in Guyana, where fisheries provide job opportunities to approximately 11,000 people [16]. In this study, most fishers fish as their sole source of income. As such, any impact on the fishing industry will directly impact their livelihoods. Most fishers were not interested in reducing bycatch as they saw it as threatening their livelihoods in a sector they claimed was declining.

Therefore, the socio-ecological linkages must be clearly understood when developing strategies to curb bycatch [34]. These must consider the sociocultural factors that exist within these linkages. Fishing traditions, knowledge, and cultural connections to communities often determine fishers’ fishing gears and methods. These are all related to the local fishing culture, and fishers generally expect some form of independence and will be reluctant to bycatch reduction measures such as gear modifications [32]. Fishers might resist changing their fishing methods to mitigate bycatch due to possible loss of income. Therefore, there is a need for education and awareness interventions for fishers and collaborative decision-making.

This study adds to the growing knowledge of bycatch composition and its impact on Guyanese fisheries. The data can provide critical insights to guide policymakers by identifying the species and life stages most affected by bycatch and the fishing practices and gear contributing to it. Although our findings demonstrate the need for sustainable fishing practices, data across small-scale fisheries in Guyana are still needed to guide decisions focusing on sustainable fisheries while maintaining coastal livelihoods.

## Supporting information

S1 Data (XLSX)
